# 3D GelMA ICC Scaffolds Combined with SW033291 for Bone Regeneration by Modulating Macrophage Polarization

**DOI:** 10.3390/pharmaceutics13111934

**Published:** 2021-11-16

**Authors:** Qian Jiang, Guo Bai, Xin Liu, Yuxiao Chen, Guangzhou Xu, Chi Yang, Zhiyuan Zhang

**Affiliations:** 1 Department of Oral Surgery, Ninth People’s Hospital, Shanghai Jiao Tong University School of Medicine, Shanghai Key Laboratory of Stomatology & Shanghai Research Institute of Stomatology, National Clinical Research Center of Stomatology, No 639, Zhizaoju Rd, Shanghai 200011, China; Jiangqian0720@hotmail.com (Q.J.); surgeonb@163.com (G.B.); xgzmy@163.com (G.X.); 2 Department of Dental Materials, Shanghai Biomaterials Research & Testing Center, Ninth People’s Hospital, College of Stomatology, Shanghai Jiao Tong University School of Medicine, Shanghai Key Laboratory of Stomatology & Shanghai Research Institute of Stomatology, National Clinical Research Center of Stomatology, No 427, Jumen Rd, Shanghai 200011, China; liuxin0556@163.com (X.L.); tennisxiaoge@163.com (Y.C.); 3 Department of Oral Maxillofacial-Head and Neck Oncology, Ninth People’s Hospital, College of Stomatology, Shanghai Jiao Tong University School of Medicine, National Clinical Research Center of Stomatology, No 639, Zhizaoju Rd, Shanghai 200011, China

**Keywords:** bone regeneration, three-dimensional gelatin-methacryloyl inverted colloidal crystal (3D GelMA ICC) scaffold, SW033291, M2 macrophage polarization, bone marrow mesenchymal stem cells (BMSCs)

## Abstract

Despite the interaction between bone marrow mesenchymal stem cells (BMSCs) and macrophages has been found to play a critical role in repairing bone defects, it remains a challenge to develop a desirable tissue engineering scaffold for synchronous regulation of osteogenic differentiation and macrophage polarization. Herein, this study proposed a novel strategy to treat bone defects based on three-dimensional Gelatin Methacryloyl Inverted Colloidal Crystal (3D GelMA ICC) scaffold and an active 15-hydroxyprostaglandin dehydrogenase (15-PGDH) inhibitor SW033291. Specifically, the 3D GelMA ICC scaffolds were firstly prepared by colloidal templating method, which displayed good cell attachment and promoted intercellular interaction among macrophage and BMSCs due to its uniform pore interconnectivity. By combined use of SW033291, the release of Prostaglandin E2 (PGE2) from BMSCs on the GelMA ICC scaffold was significantly upregulated and macrophages M2 polarization was markedly increased. In turn, BMSCs proliferation and osteogenic differentiation was further enhanced by paracrine regulation of M2 macrophage, and thus finally caused more in vivo new bone formation by shaping up a pro-regenerative local immune microenvironment surrounding GelMA ICC scaffold. Our findings demonstrate the potential of 3D GelMA ICC scaffolds combined with SW033291 to become an effective tissue engineering strategy for bone regeneration.

## 1. Introduction

Bone fractures are one of the most common injuries seen in emergency departments, with over 2 million fractures observed in the United States in 2013 [1]. Despite the best treatment efforts, about 5~10% of bone fracture cases remain undesirable outcomes because of non-union fracture [2]. An average cost of treatment of the non-united fracture has been estimated at approximately USD 11,333 in the United State [3]. Although autologous bone grafting represents the gold standard therapeutic strategy, the supply of autologous bone is insufficient and donors site morbidity [4]. Fortunately, the development of bone tissue engineering which replace a natural organ seems to be the solution to tackle the abovementioned issue [5], but it remains challenging to develop an ideal tissue engineering scaffold with excellent mechanical properties, biocompatibility, and bioactivity. The inflammatory response in the local region of the bone defect may lead to vascular occlusion, neovascularization reduction and then impede bone formation [6]. Particularly, macrophage plays a key role in the regulation of inflammation and tissue regeneration by polarizing from a resting state (M0) to a pro-inflammatory phenotype (M1) or an anti-inflammatory phenotype (M2), which can tune an inflammatory microenvironment towards a pro-regenerative niche by M2 polarization [7,8,9,10,11]. Moreover, due to some unique advantages such as the abundant sources, easy to obtain and low immunogenicity, bone marrow mesenchymal stem cells (BMSCs) with the characteristic features like self-renewal plasticity, immunoregulatory, and multi-lineage differentiation potential (differentiation into adipocyte, chondrocyte and osteocytes), has become a promising potential candidate for cellular therapies and bone tissue engineering [12,13]. Therefore, to develop a desirable tissue engineering scaffold for the regeneration of bone defects, it will be critical to modulate macrophages M2 polarization in the immune microenvironment and to promote osteogenic differentiation of bone marrow mesenchymal stem cells (BMSCs).

Recent years, due to uniformity and interconnectivity of pores in scaffolds which are suitable for diffusion of molecules (oxygen and nutrients) as well as homogeneous cell distribution, three-dimensional Gelatin Methacryloyl Inverted Colloidal Crystal (3D GelMA ICC) have been developed to fabricate scaffolds with ideal geometries and structures exhibiting precise control of pore size, porosity, and pore morphology to treat large-scale bone defects [14], eliciting greater control over cellular activities and a highly promising prospect in bone tissue engineering. Prostaglandin E2 (PGE2), a lipid signaling molecule, has been reported to accelerate M2 macrophage polarization, increase IL-10 expression, and inhibit inflammatory responses [15]. A recent study also showed that a small molecule inhibitor of 15-hydroxyprostaglandin dehydrogenase (15-PGDH) enzyme (SW033291) could increase PGE2 through inhibition of its degradation enzyme activity and then promote tissue regeneration in various tissues, including liver, intestine, and hematopoietic cells in the bone marrow [9]. However, up to now, few studies focus on how to improve the osteogenic efficiency of BMSCs laden GelMA ICC scaffolds by PGE2 mediated macrophages M2 polarization. Moreover, the mechanism underlying crosstalk between macrophages and BMSCs involved in GelMA ICC scaffolds mediated bone regeneration has not been elucidated.

**Figure 1:**
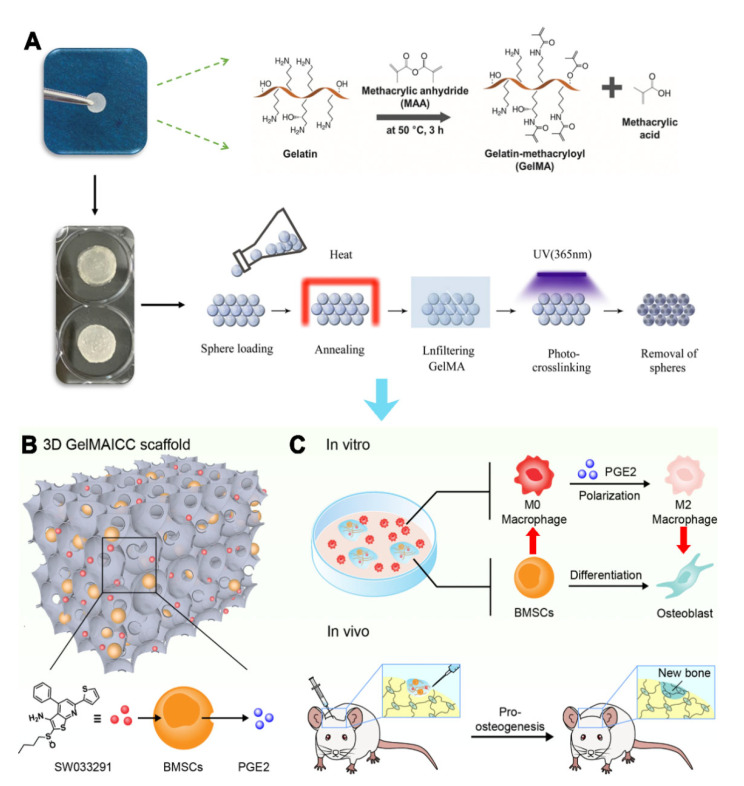
Schematic illustration of (**A**) Fabrication of GelMA ICC scaffolds with interconnected porous architectures; (**B**) PGE2 release of BMSCs mediated by SW033291; (**C**) GelMA ICC scaffolds with SW033291 promoted osteogenic differentiation by the immunoregulatory loop between BMSCs and macrophages in vitro and in vivo.

Herein, the present study proposed a novel strategy to treat bone defects based on BMSCs laden 3D GelMA ICC scaffold and PGE2 mediated macrophage polarization. Specifically, the 3D GelMA ICC scaffolds were firstly prepared by colloidal templating method, which displayed good cell attachment and promoted intercellular interaction among macrophage and BMSCs due to its uniform pore interconnectivity and high structural stability. Combining with the biofunction of SW033291 on PGE2 release of BMSCs, the 3D GelMA ICC scaffolds resulted in improved macrophage M2 polarization and osteogenic differentiation in the BMSCs. Furthermore, the GelMA ICC scaffolds with SW033291 could regulate the local microenvironment in bone defects and promote new bone regeneration in vivo (Figure 1). Our findings demonstrate the potential of 3D GelMA ICC scaffolds combined with SW033291 to become an effective tissue engineering strategy for bone regeneration applications.

## 2. Materials and Methods

### 2.1. Preparation of GelMA ICC with SW033291

GelMA ICC provided from Wenzhou Youmo Biotechnology Co., Ltd. (Wenzhou, China) were prepared according to the previously published method via colloidal templating [16]. Briefly, GelMA samples with 70%~80% degrees of substitution were synthesized according to the previously published literature [17]. Polystyrene beads of 138.1 ± 2.2 μm diameter (Duke Scientific Corporation, Palo Alto, CA, USA) were self-assembled to lattices, subsequently the 30 *w*/*v*% GelMA solution containing 1 *w*/*v*% 2-hydroxy-4′-(2-hydroxyethoxy)-2-methylpropiophenone (I2959) (Sigma-Aldrich, St Louis, MO, USA) were soaked into the lattice. After exposing to ultraviolet light for 10 min, polystyrene beads were removed from lattice using tetrahydrofuran to obtain the GelMA ICC scaffolds. The GelMA ICC scaffold has some major characteristics including 30% gelatin methacryloyl, the diameter of micropores around 135–140 µm and fast degradation in a collagenase solution (within 5–6 h). Besides, the as prepared GelMA ICC scaffolds show 1~4 kPa shear modulus. SW033291 was purchased from Selleck Chemical (S7900, Houston, TX, USA). The working concentration of sw033291 was 1 μm for combined use with gelma icc scaffolds in vitro.

### 2.2. Cell Culture and Treatment

#### 2.2.1. Isolation and Culture of Primary Bone Marrow Mesenchymal Stem Cells (BMSCs)

Six healthy 4-week-old male Sprague-Dawley rats were purchased from Shanghai Xipuer-Bikai Laboratory Animal Co., Ltd. (Shanghai, China). Bone marrow mesenchymal stem cells were isolated and cultured as previously described [18]. All rats were euthanized and immersed in 75% alcohol for 5 min and the femoral bone was then anatomized from lower limbs. The bone marrow was then harvested by a 1-mL syringe. After that, bone marrow stem cells were cultured in Dulbecco’s modified Eagle’s medium (DMEM; Gibco Life Technologies, Carlsbad, CA, USA) supplemented with 10% fetal bovine serum (FBS; Gibco, Gaithersburg, MD, USA) at 37 °C in 5% CO_2_. The P3 generation BMSCs were harvested for the experiments in this study. The CD44, CD45, CD90, and CD34 surface markers were used to identify the P3 generation primary BMSCs by flow cytometer (BD Biosciences, San Jose, CA, USA).

#### 2.2.2. Isolation and Culture of Primary Macrophages

The primary macrophages cells were isolated and cultured as previously described [19,20]. In brief, 3 mL of thioglycolate medium was injected into the intraperitoneal cavity in 3 days before the cell harvest. After that, the rat had been injected PBS (Phosphate Buffer Saline) solution into the peritoneal cavity. The liquid in the abdominal cavity was gently collected by 3-mL syringe centrifuging at 1000 r/min at 4 °C for 10 min. After incubating in 5% CO_2_ for 2 h, the medium was changed and washed 1–2 times with RPMI1640 medium. The IL-6, TNF-α, and CD206 antibodies were used to determine the surface markers of primary macrophages, M1 macrophages and M2 macrophages, by flow cytometry.

#### 2.2.3. Cell Treatment

The 3D GelMA ICC scaffolds were sterilized by 70% ethanol and immersed in PBS at 37 ℃ for 1–2 h and then centrifuged at 2500–3000 rpm for 10–15 min to remove bubbles within scaffolds. Subsequently, the 3D GelMA ICC scaffolds were carefully transferred into a 24 well plate using tweezers. After BMSCs (100,000 cells) were seeded, the scaffolds were placed in CO_2_ incubator for 3–4 h for further cell attachment. For the coculture model, BMSCs seeded on 3D GelMA ICC scaffolds were placed onto the bottom of 24-well culture plates and the primary macrophages (100,000 cells) were cultured on Transwell inserts (Millipore; Billerica, MA, USA) in the plates. All in vitro experiments were performed three times with each individual experiment carried out in triplicate.

### 2.3. Cell Viability Assay

To evaluate the effect of 3D GelMA ICC scaffolds on BMSCs growth, the cell viability assays were performed by cell counting kit-8 (CCK-8) assay (Beyotime, Shanghai, China) and Calcein-AM (Life Technologies, C1430) + PI (Sigma P4170) fluorescent dyes on days 1, 3, 5, and 7 of BMSCs cultured on 3D GelMA ICC scaffolds according to the manufacturer’s instructions. To further investigate the effect of macrophages and SW033291 on cell viability, the BMSCs cultured on the 3D GelMA ICC scaffolds for 5 days were served as the control group. After co-culture with the primary macrophages (M0 macrophage) in the presence and in the absence of SW033291 (1 μM) for 5 days, the BMSCs on the scaffolds were washed two times with PBS and then were stained with Calcein-AM + PI at 37 °C for 30 min. After rinsing twice with PBS, they were analyzed by fluorescence microscopy (Leica, Heidelberg, Germany). The cell viability was calculated by ImageJ software v1.8.0.

### 2.4. Assessment of Macrophage Polarization

To investigate the effect of 3D GelMA ICC scaffolds, BMSCs and SW033291 on macrophage polarization, the M0 macrophage co-cultured with BMSCs were served as the control group. After co-culture with BMSCs on 3D GelMA ICC scaffolds in the presence and in the absence of SW033291 (1 μM) for 5 days, the polarization of macrophage cells determined by flow cytometry with the expression levels of IL-6 (M1 marker) and CD206 (M2 marker). The cell supernatants were collected for further ELISA analysis. Briefly, the macrophage cells were fixed with 4% paraformaldehyde for 15 min, permeabilized with 0.1% Triton-X for 30 min, and blocked using 5% bovine serum albumin (BSA) for 1 h. Samples were incubated with IL-6 Antibody (11-7069-82) or anti CD206 antibody (MMR) Antibody (17-2061-82) at 4 °C and then with secondary Alexa Fluor 594 antibodies (1:500; Abcam, Cambridge, UK) for 2 h at room temperature. Finally, the proportion of cells expressing IL-6 and CD206 in the different experimental conditions was determined with flow cytometry.

### 2.5. Enzyme-Linked Immunosorbent Assay (ELISA)

The release of prostaglandin PGE2, IL-1β and CD206 in the above supernatants were assessed by ELISA. The levels of IL-1β, PGE2 and CD206 in different groups were detected according to the manufacture’s instruction (Shanghai Jingkang Biological Engineering Co., Ltd., Shanghai, China). Briefly, 96 well flat bottom plates were used for coating with PGE2, IL-1β and CD206 polyclonal antibody, respectively. Plates were blocked for 1 h at room temperature with 200 uL of filtered 4% BSA in DPBS. For ELISA assays, recombinant PGE2, IL-1β and CD206 standards were run with 1:2 serial dilutions. Streptavidin-HRP (R&D Systems, DY998, Minneapolis, MN, USA) antibody was used, and ELISA plates were developed with SureBlue TMB Microwell Peroxidase Substrate (KPL, 52-00-00). TMB Stop Solution (KPL, 50-85-05) was added to halt the reaction. Dilutions for standard, detection antibody, and streptavidin-HRP were in filtered 4% BSA in DPBS. All washes were done using a BioTek ELx405 plate washer with 0.05% Tween-20 in DPBS. The absorbance at 450 nm was measured on a Molecular Devices FlexStation 3 Reader (Molecular Devices, Sunnyvale, CA, USA).

### 2.6. Protein Detection by Western Blotting

After co-culture with the primary macrophages (M0 macrophage) in the presence and in the absence of SW033291 (1 μM) for 5 days, the BMSCs on the scaffolds were harvested and protein extracts were prepared for Western blot. The BMSCs cultured on the 3D GelMA ICC scaffolds for 5 days were set as the control group. Cells were harvested and lysed on ice in RIPA Lysis Buffer (Beyotime Institutte of Biotechnology, Jiangsu, China). A bicinchoninic acid assay (Thermo Fisher Scientific, Inc., Waltham, MA, USA) was performed to measure the concentration of protein. Equal quantities of protein (50 µg) were loaded on an SDS gel, resolved using SDS-PAGE and transferred to a PVDF membrane. Membranes were blocked in 5% skimmed milk for 2 h. Subsequently the membranes were incubated with primary antibodies against these primary antibodies that as follows: primary antibodies against OCN (Osteocalcin) (ab93876, 1:1000), Runx2 (Runt-related transcription factor 2) (ab92336, 1:1000); ALP (Alkaline phosphatase) (ab224335, 1:1000); a-tubulin (ab7291, 1:1000) from Abcam Plc, Cambridge, UK overnight at 4 °C. Subsequently, the membranes were washed three times with PBS containing 0.05% Tween-20 and incubated with the secondary antibody (goat anti-rabbit IgG, 1:10,000; Licor) at room temperature for 2 h. The membranes were washed again, and target bands and detected using an enhanced chemiluminescence reagent (Thermo Fisher Scientific, Inc., Waltham, MA, USA) and analyzed using ImageJ software v1.8.0 (National Institutes of Health, New York, NY, USA). a-tubulin was used as the internal control. The experiments were repeated three times.

### 2.7. Morphology Characterization

Surface morphology of the BMSCs + GelMA ICC scaffolds co-culture in different groups on day 0, day 3, day5 and day 7 was examined by scanning electron microscopy (JSM-7600F, JEOL, Tokyo, Japan). These samples were fixed with electron microscopic fixator, and then were dehydrated with 25%, 50%, 75%, 95%, and 100% ethanol for 15 min respectively. GelMA ICC scaffolds was stored at 80 and lyophilized for 48 h. The scanning electron microscopy images were estimated by ImagePro-Plus 6.0^®^ software.

### 2.8. Alizarin Red Staining

After co-culture with the primary macrophages (M0 macrophage) in the presence and in the absence of SW033291 (1 μM) for 21 days, the BMSCs + GelMA ICC, BMSCs + GelMA ICC + M0 and BMSCs + GelMA ICC + SW0332891 groups were fixed with 4% paraformaldehyde for 15 min and blocked with 1% BSA for 1 h. Alizarin red staining was then performed by Alizarin red staining kit (Genmed, Shanghai, China) respectively according to the manufacturer’s instructions.

### 2.9. Experimental Calvarial Rat Model

All animal experiments were performed in accordance with the Guidelines for the Care and Use of Laboratory Animals. The rat calvarial model was established according to a method reported by Spicer et al. [21]. To prepare the GelMA ICC scaffold with SW033291 for in vivo use, the GelMA ICC scaffold was totally immersed with the SW033291 drug solution (50 mM) in 100 μL DMSO to absorb 2 mg SW033291 per scaffold. Forty rats were divided into 4 groups including the sham group, the GelMA ICC group in which the defect was treated with GelMA ICC alone, the BMSCs + GelMA ICC group in which the defect was treated with BMSCs laden GelMA ICC scaffolds, and the BMSCs + GelMA ICC + SW033291 group in which the defect was treated with BMSCs laden GelMA ICC scaffold with SW03329. Briefly, all rats were anesthetized with an intra-peritoneal injection of 1% pentobarbital (10 μL g^−1^). Next, the head skin was sterilized with povidone iodine, and a 5 mm diameter calvarial defect was made carefully in both sides. The holes at right side were treated with GelMA ICC alone or BMSCs + GelMA ICC or BMSCs + GelMA ICC + SW033291, while the left side holes were treated by Sham or GelMA ICC as the control. Rats were given free access to water and food and they were sacrificed at 4 weeks and 8 weeks after calvarial experiment.

### 2.10. Micro-Computed Tomography (Micro-CT)

The rats in different groups were sacrificed, and skulls were removed at 4 weeks and 8 weeks respectively. For evaluation of bone remodeling, the skulls were scanned with an in vivo micro-CT system (SkyScan 1176, Bruker, Karlsruhe, Germany). After scanning, 3D images were analyzed and the bone volume fractions (BV/TV) were calculated by auxiliary software.

### 2.11. Hematoxylin and Eosin (H&E) and Manson Staining

After micro-CT examination, calvarial specimens in different groups were fixed with 4% paraformaldehyde and embedded in paraffin. After that the sections were cut a thickness of 5 μm and stained with H&E and Manson stains. The slices were analyzed by light microscopy.

### 2.12. Statistical Analysis

Statistical analysis was performed with SPSS version 19.0 (IBM, Armonk, IL, USA). All results are shown as means ± standard deviations. Results were analyzed by independent *t*-tests and one-way analysis of variance. Results with *p* values of less than 0.05 were considered significant.

## 3. Results

### 3.1. Effects of GelMA ICC on BMSCs Viability

**Figure 2:**
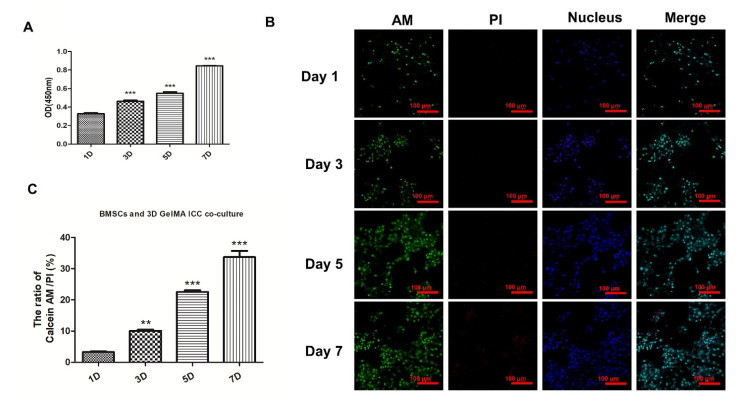
Effects of GelMA ICC scaffold on BMSCs viability. (**A**) The cell viability of BMSCs seeded on GelMA ICC scaffolds detected by CCK-8; (**B**) The cell viability of BMSCs seeded on GelMA ICC scaffolds detected by Calcein AM (live cells in green)/PI (dead cells in red); (**C**) The ratios of Calcein AM/PI (live cells vs. death cells); Data represent mean ± standard deviation, *n* = 3 (** *p* < 0.05; *** *p* < 0.01 vs. 1 day group).

The effects of GelMA ICC on BMSCs viability were firstly identified by Calcein-AM/PI live/dead cell double staining kit and CCK-8 assay at preset time point. The OD values of BMSCs seeded on GelMA ICC scaffolds for 3, 5, and 7 days were significantly higher than the group for 1 day (*p* < 0.01), indicating a time-dependent increase trend on cell viability (Figure 2A). Moreover, the results of Live/Dead assays showed that viable cells (green fluorescence) were dominant on surface of GelMA ICC scaffolds for day 3 and day 5, and till the 7th day, a few of apoptotic cells (red fluorescence) had appeared. These results showed that the GelMA ICC scaffold exhibited satisfactory biocompatibility.

### 3.2. Effect of GelMA ICC with SW033291 on the Polarization of Macrophages

**Figure 3:**
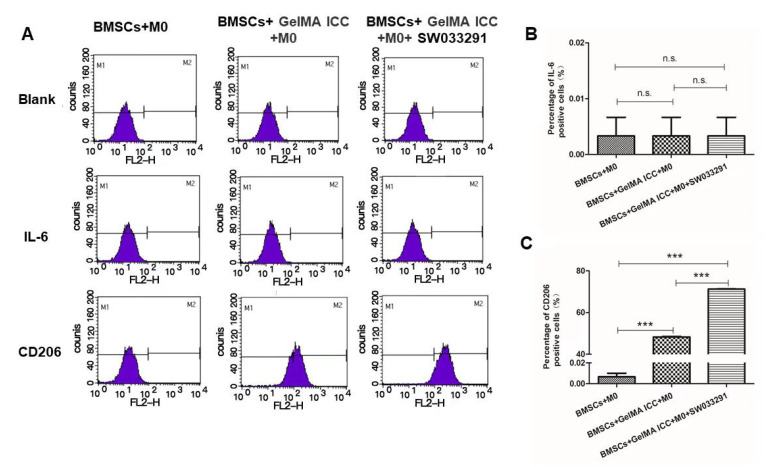
Effect of GelMA ICC with SW033291 on the polarization of macrophages (**A**) Histogram of flow cytometry analysis showing the expressions of markers of macrophages polarization including M1 (IL6) and M2 (CD206) in different groups; (**B**) The percentage of IL-6 and CD206 positive cells (n.s: no significance); (**C**) The percentage of CD206 positive cells. Data represent mean ± standard deviation, *n* = 3. (*** *p* < 0.01 vs. the BMSCs + M0 control group).

As shown in Figure 3, we performed flow cytometry in vitro to evaluate the expression of M1 (IL6) and M2 (CD206) markers in macrophages in the BMSC + M0, BMSCs +GelMA ICC + M0 and BMSCs + GelMA ICC + M0 + SW033291 groups by using the non-contact coculture system. The results indicated that only BMSCs co-cultured with M0 macrophages had no effect on polarization of macrophages, and the IL-6 and CD206 expression were negative. The percentage of positive cells expressing CD206 in macrophage of co-cultures in GelMA ICC with SW033291 group was significantly higher than that in the GelMA ICC group, while the IL-6 expression was not induced by GelMA ICC with or without SW033291 treatment, showing that GelMA ICC combined with SW033291 were able to regulate the M2 macrophage polarization in the co-culture with BMSCs system.

### 3.3. Effects of GelMA ICC with SW033291 on the Cell Viability, Morphologies and Differentiation of BMSCs in Co-Culture

**Figure 4:**
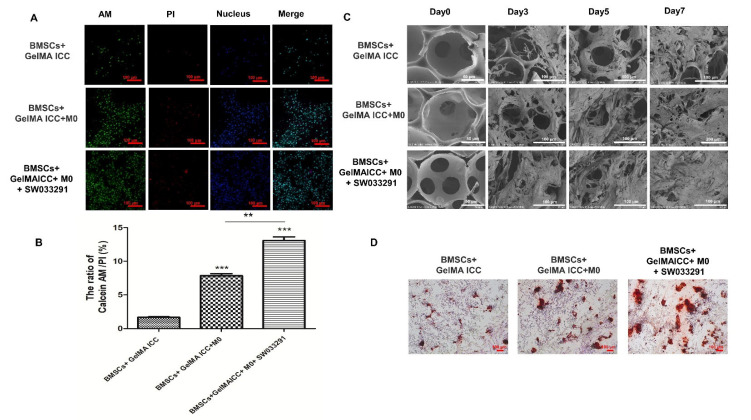
Effects of GelMA ICC with SW033291 on the cell viability, morphologies, and differentiation of BMSCs in co-culture. (**A**) The cell activity of BMSCs in coculture treated with GelMA ICC/SW033291 detected by Calcein AM (live cells in green)/PI (dead cells in red); (**B**) The ratios of live cells and death cells; (**C**) Characterization of BMSCs morphologies in GelMA ICC scaffolds during the culture in different groups; (**D**) The images of the Alizarin red staining in different groups. Data represent mean ± standard deviation, *n* = 3. The BMSCs seeded on GelMA ICC was served as the control, ** *p* < 0.05 significant difference as compared groups. *** *p* < 0.01 vs. the control group.

To identify whether the GelMA ICC with SW033291 can promote the BMSCs viability and the polarization of M0 macrophages towards M2 type during the bone formation, the non-contact co-culture of the M0 macrophages and the BMSCs laden GelMA ICC scaffold were conducted, and the double staining of Calcein-AM/PI live/dead cells was performed to observe cell viability (Figure 4A). The results showed that cell viability increased significantly in the BMSCs + GelMA ICC and BMSCs + GelMA ICC + SW033291 groups than that in the BMSCs groups (Figure 4C, *p* < 0.01). The results indicated that GelMA ICC combined with SW033291 could be benefit for BMSCs growth in the BMSCs/macrophage co-culture systems.

The scanning electron microscope images exhibit the clear morphological differences between the BMSCs grown in GelMA ICC and those grown on GelMA ICC + M0 and GelMA ICC + M0 + SW033291 substrates on days 3, 5, and 7. On the 3rd day, BMSCs in GelMA ICC scaffolds initially anchored to the cavity walls and gradually grew into the cell sheet constructs. On the 5th day, this trend become more obvious. On the 7th days, most of the cavities were filled with cells which then expanded to form multi-cell clusters in the GelMA ICC + M0 and GelMA ICC+ M0 + SW033291 groups. The highest cell density was observed in M0 + GelMA ICC+ SW033291 and followed by those in the GelMA ICC + M0 and GelMA ICC groups (Figure 4B).

The Alizarin red staining were used to determine osteogenic differentiation and mineralized nodule formation in the BMMSCs. Here, more mineralized nodule formation was observed in the BMSCs of coculture treated with GelMA ICC and SW033291 than the GelMA ICC group and the negative control, showing GelMA ICC with SW033291 could effectively promote osteogenic differentiation of BMSCs (Figure 4D).

### 3.4. Effects of GelMA ICC with SW033291 on the Cytokines Release in Coculture

**Figure 5:**
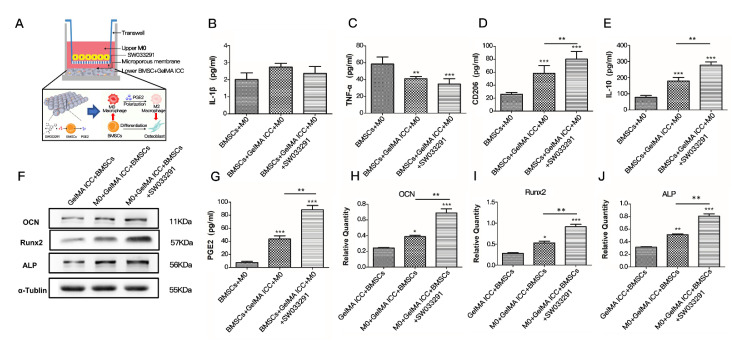
Effects of GelMA ICC scaffolds with SW033291 on the cytokines release and osteogenic pathway. (**A**) Schematic illustration of the crosstalk between BMSCs and macrophage in non-contact coculture system; (**B**) The IL-1β levels in coculture; (**C**) The TNF-α levels in coculture; (**D**)The CD206 levels in coculture; (**E**) The IL-10 levels in coculture; (**F**) Western blot of the OCN, Runx2 and ALP levels in BMSCs of co-cultures with different treatment; (**G**) The PGE2 levels in coculture; (**H**) The relative density of OCN band by gray value analysis; (**I**) The relative density of Runx 2 band by gray value analysis; (**J**) The relative density of ALP band by gray value analysis. Data represent mean ± standard deviation, *n* = 3. For cytokines release, the BMSCs + M0 coculture was served as the control. For Western blot analysis, the BMSCs seeded on GelMA ICC was served as the control, * *p* < 0.05 significant difference as compared groups. ** *p* < 0.05 significant difference as compared groups. *** *p* < 0.01 vs. the control group.

After 5 days, BMSCs/macrophage co-cultures in the GelMA ICC with SW033291 group exhibited more PGE2 protein secretion than that in GelMA ICC alone group and the negative control group (Figure 5G), PGE2 level in GelMA ICC group was significantly higher than that in the BMSCs/macrophage coculture group (*p* < 0.05).

The expression levels of inflammatory cytokines including TNF-α and IL-1β and anti-inflammatory factors, including IL-10 and CD 206 were assessed by ELISA assay. As shown in Figure 5B,C, the pro-inflammatory cytokines (TNF-α and IL-1β) were not significantly induced in all groups. In contrast, the anti-inflammatory cytokines (IL-10, CD 206) were significantly upregulated in the coculture system treated with GelMA ICC alone or GelMA ICC with SW033291, and more IL-10 and CD206 release were observed in the GelMA ICC with SW033291 group (*p* < 0.05) (Figure 5D,E), showing that GelMA ICC with SW033291 could induce M2 type polarization in macrophages and release anti-inflammatory cytokines.

### 3.5. Effects of GelMA ICC Scaffolds with SW033291 on the Osteogenic Pathway

To further evaluate the effects of GelMA ICC with SW033291 on the osteogenic pathway, we performed western blot to detect the expression of Runx2, OCN and ALP. As shown in Figure 5H,J, Runx2, OCN, and ALP expression levels were the highest in the BMSCs of coculture with M0 in the GelMA ICC with SW033291 group (*p* < 0.05), followed by those in the BMSCs of coculture with M0 in the GelMA ICC group and then those in the BMSCs of monoculture in the GelMA ICC group.

### 3.6. Effect of GelMA ICC Scaffolds with SW033291 on the Bone Formation In Vivo

**Figure 6:**
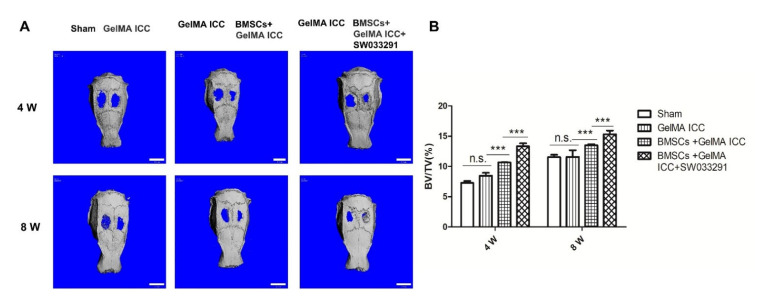
Micro-CT showed the new bone formation in each group at 4 and 8 weeks after surgery (scale bar: 5 mm). (**A**) Reconstruction of micro-CT images; (**B**) Quantitative analysis of micro-CT images including BV/TV. Data are represented as mean ± standard deviation, *n* = 5 *** *p* < 0.01.

**Figure 7:**
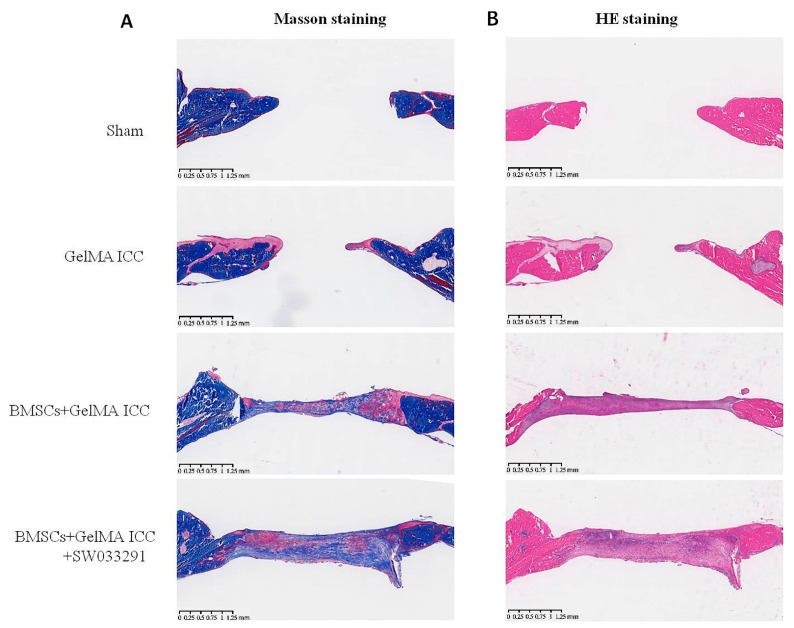
Histological staining for bone formation at the calvarial region in different groups. (**A**) Masson staining and (**B**) H&E staining for new bone formation.

In our in vivo study, we established a 5 mm calvarial critical-size defect rat model. After 4 and 8 weeks of implantation, micro-CT was used to observe the new bone formation by quantitatively analysis. The results confirmed that the indexes reflected new bone formation (Figure 6). We found that bone volume fraction (BV/TV) of BMSCs + GelMA ICC + SW033291 group was significantly higher than that of BMSCs + GelMA ICC and GelMA ICC groups, indicating better bone regeneration performance. The bone formations in the calvarial regions were also detected by HE staining and Manson staining. Figure 7 showed both new bone and new blood vessel formation were increased in the BMSCs +GelMA ICC + SW033291 group and the BMSCs + GelMA ICC groups than that in the Sham and the GelMA ICC groups. The new bone volume and degree of neovascularization were highest in the BMSCs + GelMA ICC + SW033291 group, followed by those in the BMSCs +GelMA ICC and GelMA ICC groups.

## 4. Discussion

Bone defect healing is known to a complex and dynamic process modulated by the interaction of multiple cells (e.g., osteoblasts, osteoclasts, macrophages, endothelial cells) and extracellular matrix [11]. Among these cells, macrophages play a critical role in the entire bone healing process due to its high plasticity (M1/M2 polarization) for regulation of the immune microenvironment surrounding bone defects. At the early stages of bone healing, M1 macrophages contribute to the restoration of tissue homeostasis by amplifying the inflammatory reaction and recruiting immune cells. At later stages of bone healing, M2 macrophages contribute to tissue regeneration by secreting anti-inflammatory cytokines and growth factors [22,23,24]. Therefore, the present study successfully developed a novel strategy based on 3D GelMA ICC scaffold and SW033291 release which can simultaneously promote the osteogenic differentiation of BMSCs and modulate macrophage towards M2 phenotypes for bone regeneration both in vitro and in vivo.

GelMA ICC scaffolds are the new promising materials possessing highly organized interconnected porous architectures and tunable biodegradation properties for tissue engineering [16]. In the present study, to closely mimic the microenvironment of the bone extracellular matrix, a 3D GelMA ICC scaffold was firstly prepared using 30% gelatin-methacryloyl with the degree of substitution around 70%~80%, which displayed their micropores with a diameter of around 135–140 µm and showed 1~4 kPa shear modulus. Moreover, it could degrade fast (within 5–6 h) in a collagenase solution (1 mg/mL in HBSS buffer). As is well known, the tissue engineering scaffolds with capacity for promoting cell adhesion and growth of stem cells is a prerequisite for successful repair of bone defect [25,26,27]. Our cells viability studies demonstrated that BMSCs grew well and increasingly on the 3D GelMA ICC scaffold at 1, 3, 5, and 7 days and the cells actively proliferated levels significantly higher during 3 to 7 days (Figure 1), showing GelMA ICC scaffold could provide favorable growth environments for BMSCs proliferation. This is consistent with the other studies, which showed that hepatocytes loaded in 3D GelMA ICC scaffolds could help cells better maintain viability and functionality by cell–cell and cell–ECM interactions between interconnected pores over the 9-day culture period [16].

Besides the direct facilitating effects of scaffold materials on BMSCs growth, the interaction between BMSCs and macrophages have drawn more attention in scaffold-based tissue engineering [15]. The key role of macrophages in the differentiation of mesenchymal stem cells during bone regeneration has been indicated. Indeed, animal studies have comprehensively demonstrated that fractures do not heal without the direct involvement of macrophages [15]. Generally, macrophages are categorized into pro-inflammatory M1 (or ‘classically activated’) and anti-inflammatory M2 (or ‘alternatively activated’) phenotypes of polarization [11]. It has been found that mesenchymal stem cells could induce macrophages M2 polarization by producing soluble factors such as PGE2 under inflammatory conditions [28]. Thus, to modulate the macrophage into anti-inflammatory M2 phenotype, a small molecule inhibitor (SW033291) of 15-PGDH, which previously increased prostaglandin PGE2 levels in bone marrow tissues [15], was combined with 3D GelMA ICC scaffolds in this study. A non-contact coculture model of BMSCs and M0 macrophage was established to determine the effect of BMSCs laden GelMA ICC scaffold in the presence of SW033291 on macrophage polarization. Our flow cytometry results showed GelMA ICC scaffold could obviously promote the polarization of macrophages from an M0 to an M2 phenotype in the non-contact coculture compared with the control without GelMA ICC scaffold. Moreover, GelMA ICC scaffold with SW033291 could enhance the M2 polarization compared with GelMA ICC alone in this coculture system (Figure 3C). By contrast, the hallmark of M1 phenotype was no detectable in all groups (Figure 3B).

Have shown that GelMA ICC scaffold combined with SW033291 could shape up a pro-regenerative immune microenvironment, we next investigate whether the cells attachment, growth, and osteogenic differentiation of BMSCs could be enhanced in response to activated M2 macrophage polarization. SEM showed that BMSCs in GelMA ICC scaffolds initially anchored to the cavity walls and gradually grew into the cell sheet constructs over 7 days, which could be further improved by coculture with M0 macrophage and addition of SW033291 (Figure 4B). The double staining of Calcein-AM/PI live/dead cells further verified that BMSCs cultured on the GelMA ICC scaffold with M0 macrophage and SW033291 exhibited the highest cell seeding efficiency and excellent cell adhesion among all groups (Figure 4A,C). Moreover, mineral deposition of BMSCs cultured in scaffolds was analyzed by AS-R assay. Our data showed that GelMA ICC scaffolds alone could induce BMSCs to produce a few red dye calcium nodules after 21 days, and more calcium nodules were observed when coculture with M0 macrophage. When the SW033291 was added, the most calcium mineral deposits were formed (Figure 4D), which further proved that GelMA ICC combined with SW033291 could be benefit for BMSCs proliferation and osteogenic differentiation in response to macrophage M2 polarization.

BMSCs is also proven to contribute to build up an optimal microenvironment for osteoblastic maturation by macrophage recruitment and immunomodulation at the early stage of implantation [22,29]. To attain an in-depth understanding of the crosstalk between macrophages and BMSCs involved in GelMA ICC mediated bone regeneration, cytokines release in the non-contact coculture system with different treatment were subsequently determined in this study. Among cytokines released from MSCs, Prostaglandins E2 (PGE2) has been demonstrated to stimulate both bone resorption and bone formation, thus increasing bone mass and bone strength [30]. In fact, our study showed that GelMA ICC could slightly increase PGE2 levels in the BMSCs and co-treatment with GelMA ICC and SW033291 could significantly induce BMSCs to produce more PGE2 than GelMA ICC alone, suggesting the PGE2 release of BMSCs was majorly attributed to the action of small molecule inhibitor of 15-PGDH (SW033291) (Figure 5G). SW033291 as an active 15-PGDH inhibitor has been previously reported to increase prostaglandin PGE2 levels in bone marrow and other tissues and markedly potentiate tissue repair in vivo [15]. The interactions between PGE2 and M2 macrophages were associated with anti-inflammation, and mineralization during bone formation [31]. The PGE2 could stimulate M2 macrophages in the regulation of anti-inflammation during bone healing [32,33]. Previous studies have also reported that the therapeutic benefits of MSCs on colitis could be enhanced by IGF-1C hydrogel through promoting PGE2-mediated M2 macrophage polarization [34]. Consistently, our results also indicated that the hallmark of M2 macrophage polarization (IL-10, CD206) was significantly upregulated by co-treatment of GelMA ICC with SW033291, when compared with GelMA ICC alone and the negative control group (Figure 5D,E), while neither the GelMA ICC alone nor cotreatment with SW033291 did not induce the release of typical proinflammatory cytokines of M1 macrophage polarization (TNF-α,IL-1β) (Figure 5B,C), showing that the increase of M2 macrophages polarization was largely dependent on the upregulation of PGE2 in BMSCs loaded the GelMA ICC scaffolds treated with SW033291. In turn, M2 macrophages in this coculture system was also found to increase the expression of proteins in osteogenic differentiation related pathway (Runx2, OCN, ALP). Taken together with above results, our findings suggest that GelMA ICC scaffold could promote cells attachment, growth and osteogenic differentiation of BMSCs due to its organized interconnected porous architectures, when SW033291 were added, the release of PGE2 from BMSCs subsequently promoted macrophages M2 polarization which in turn enhanced BMSCs proliferation and osteogenic differentiation by paracrine regulation, and thus finally resulted in osteogenesis for bone repair and regeneration by an immunoregulatory crosstalk between BMSCs and macrophages under GelMA ICC scaffold mediated local microenvironment (Figure 5A).

Encouraged by the data acquired in vitro, we subsequently investigated the effects of BMSCs laden GelMA ICC scaffolds combined with SW033291 on repairing bone defects in vivo. A previous study indicated that GelMA ICC could promote new bone formation and neovascularization in the calvarial regions [35]. In agreement with previous research, we also showed that new bone volume was higher in the GelMA ICC group than the Sham group after the scaffold was implanted for 4 weeks and 8 weeks. Moreover, the BMSCs laden GelMA ICC scaffold combined with SW033291 group showed the highest amount of new bone volume, indicating the best osteogenic properties, followed by those in the BMSCs laden GelMA ICC group and then GelMA ICC alone group (Figure 6). Furthermore, we performed H&E staining and Masson staining to observe new bone growth in the calvarial region. Both new bone formation and neovascularization were found at the bone defect region in the BMSC + GelMA ICC + SW033291 andBMSC + GelMA ICC groups (Figure 7). These findings confirmed that the addition of SW033291 could obviously improve the osteogenic properties of GelMA ICC scaffolds in vitro and in vivo.

There are several limitations to be considered in the present study. First, the major limitation of this study is the use of immersion method to combine the SW033291 and the GelMA ICC scaffold, instead of developing a controllable drug-releasing GelMA ICC scaffold, which might result in unpredictable drug release profile during bone formation in vivo. Second, the macrophage cytokines were evaluated only on the fifth day, more time points might be more suitable for the evaluation of time-efficacy relationship. Third, it would be more important to investigate the macrophage M1/M2 polarization changes in vivo, which make our conclusion on macrophage M2 polarization and bone formation more accurate.

## 5. Conclusions

In summary, we successfully constructed a BMSCs laden 3D GelMA ICC scaffold with an active 15-PGDH inhibitor SW033291 for bone regeneration. Our findings suggest that GelMA ICC scaffold alone promoted cells attachment, growth, and osteogenic differentiation of BMSCs due to its organized interconnected porous architectures. By combined use of SW033291, the release of PGE2 from BMSCs on the GelMA ICC scaffold was significantly upregulated and macrophages M2 polarization was markedly increased. In turn, BMSCs proliferation and osteogenic differentiation was further enhanced by paracrine regulation of M2 macrophage, and thus finally caused more in vivo new bone formation by shaping up a pro-regenerative local immune microenvironment surrounding GelMA ICC scaffold. This approach provides a novel strategy for the effective combined application of 3D GelMA ICC scaffolds and 15-PGDH inhibitor SW033291 in the bone tissue engineering field.

## Data Availability

All data in this study have been included in this manuscript.
